# Pregnancy Outcomes among Pregnant Persons after COVID-19 Vaccination: Assessing Vaccine Safety in Retrospective Cohort Analysis of U.S. National COVID Cohort Collaborative (N3C)

**DOI:** 10.3390/vaccines12030289

**Published:** 2024-03-11

**Authors:** Emily A. G. Faherty, Kenneth J. Wilkins, Sara Jones, Anup Challa, Qiuyuan Qin, Lauren E. Chan, Courtney Olson-Chen, Jessica L. Tarleton, Michael N. Liebman, Federico Mariona, Elaine L. Hill, Rena C. Patel, The N3C Consortium

**Affiliations:** 1 Division of Epidemiology and Community Health, University of Minnesota School of Public Health, Minneapolis, MN 55455, USA; 2 Biostatistics Program, Office of the Director, National Institute of Diabetes and Digestive and Kidney Diseases, National Institutes of Health, Bethesda, MD 20892, USA; kenneth.wilkins@nih.gov; 3 Office of Data Science and Emerging Technologies, National Institute of Allergy and Infectious Diseases, National Institutes of Health, Rockville, MD 20892, USA; jonessarae@gmail.com; 4 Vanderbilt Institute for Clinical and Translational Research, Vanderbilt University Medical Center, Nashville, TN 37203, USA; anup.p.challa@vanderbilt.edu; 5 Department of Public Health Sciences, University of Rochester Medical Center, Rochester, NY 14642, USA; qiuyuan_qin@urmc.rochester.edu (Q.Q.); elaine_hill@urmc.rochester.edu (E.L.H.); 6 Department of Pediatrics, University of Chicago, Chicago, IL 60637, USA; 7 Department of Obstetrics and Gynecology, University of Rochester Medical Center, Rochester, NY 14620, USA; courtney_olson-chen@urmc.rochester.edu; 8 Department of Obstetrics and Gynecology, Medical University of South Carolina, Charleston, SC 29425, USA; tarletonj@musc.edu; 9 IPQ Analytics, LLC, Kennett Square, PA 19348, USA; michael.liebman@ipqanalytics.com; 10 Beaumont Hospital, Dearborn, MI 48124, USA; fmariona@att.net; 11 School of Medicine, Wayne State University, Detroit, MI 48201, USA; 12 Departments of Medicine and Global Health, University of Washington, Seattle, WA 98195, USA; renapatel@uabmc.edu; 13 Department of Medicine, University of Alabama at Birmingham, Birmingham, AL 35233, USA

**Keywords:** COVID-19 vaccination, pregnancy, safety, preterm birth, stillbirth, U.S., real-world data

## Abstract

COVID-19 vaccines have been shown to be effective in preventing severe illness, including among pregnant persons. The vaccines appear to be safe in pregnancy, supporting a continuously favorable overall risk/benefit profile, though supportive data for the U.S. over different periods of variant predominance are lacking. We sought to analyze the association of adverse pregnancy outcomes with COVID-19 vaccinations in the pre-Delta, Delta, and Omicron SARS-CoV-2 variants’ dominant periods (constituting 50% or more of each pregnancy) for pregnant persons in a large, nationally sampled electronic health record repository in the U.S. Our overall analysis included 311,057 pregnant persons from December 2020 to October 2023 at a time when there were approximately 3.6 million births per year. We compared rates of preterm births and stillbirths among pregnant persons who were vaccinated before or during pregnancy to persons vaccinated after pregnancy or those who were not vaccinated. We performed a multivariable Poisson regression with generalized estimated equations to address data site heterogeneity for preterm births and unadjusted exact models for stillbirths, stratified by the dominant variant period. We found lower rates of preterm birth in the majority of modeled periods (adjusted incidence rate ratio [aIRR] range: 0.42 to 0.85; *p*-value range: <0.001 to 0.06) and lower rates of stillbirth (IRR range: 0.53 to 1.82; *p*-value range: <0.001 to 0.976) in most periods among those who were vaccinated before or during pregnancy compared to those who were vaccinated after pregnancy or not vaccinated. We largely found no adverse associations between COVID-19 vaccination and preterm birth or stillbirth; these findings reinforce the safety of COVID-19 vaccination during pregnancy and bolster confidence for pregnant persons, providers, and policymakers in the importance of COVID-19 vaccination for this group despite the end of the public health emergency.

## 1. Introduction

COVID-19 vaccination is one of the most effective tools against severe morbidity and mortality in the global COVID-19 pandemic. COVID-19 vaccines have demonstrated very high clinical efficacy in registrational trials, particularly among mRNA vaccines produced by Pfizer–BioNTech (BNT162b2) (95%) and Moderna (mRNA-1273) (94%) [1,2]. Post-marketing studies of vaccine effectiveness demonstrate that these vaccines have protected against death and hospitalization in all phases of the pandemic, including the most recent Omicron-dominant period [3,4,5]. While pregnant persons were not systematically included in most registrational trials, real data corroborate high COVID-19 vaccine effectiveness in this population [6,7]. Active immunization has been shown to be protective against both infection and hospitalization in pregnant persons [8,9].

Vaccine-produced immunity in pregnancy may be longer lasting than infection-induced immunity, and pregnant persons have mounted immune responses as robust as those of persons who are not pregnant [10,11]. Furthermore, vaccination during pregnancy may reduce the likelihood of adverse pregnancy outcomes associated with COVID-19 infection itself, such as preterm birth and non-reassuring fetal monitoring [12]. However, few studies have accounted for differences in the predominant circulating COVID-19 variants themselves [13], noting overall less severe illness during the Omicron wave compared to earlier periods [14]. An examination of the effects of vaccination on pregnancy based on the predominant variant periods can suggest whether temporal relationships contribute to associations purported between vaccination and adverse pregnancy outcomes.

Given the novel nature of SARS-CoV-2 infection and the relatively rapid development and deployment of COVID-19 vaccines, evidence of vaccine safety in the implementation setting remains necessary in order to recommend vaccination among vulnerable groups, such as pregnant persons. Based on data from completed clinical trials and post-marketing experience largely driven by spontaneous safety case reports, most adverse maternal or neonatal outcomes occur uncommonly and, therefore, would be best examined with respect to healthcare implementation in large datasets derived from real-world settings. Using a large, nationally sampled electronic health record (EHR) repository, we aimed to estimate the association between COVID-19 vaccination and both preterm birth and stillbirth, which are adverse pregnancy outcomes that can be identified using EHR. We hypothesized that COVID-19 vaccination will not be associated with a greater risk of these adverse pregnancy outcomes.

## 2. Materials and Methods

### 2.1. Data Sources and Overall Structure

This study used data from the National COVID Cohort Collaborative (N3C), a national platform of aggregated and harmonized de-identified EHR data from over 70 healthcare systems across the United States for use in COVID-19 research [15,16]. Data partner sites contribute demographic, visit, vital status, medication, laboratory, diagnoses, and imaging data to a central data repository that is harmonized on a regular basis according to the Observational Medical Outcomes Partnership (OMOP) common data model (CDM). The N3C is supported by the National Center for Advancing Translational Science (NCATS) of the National Institutes of Health (NIH), and data and supporting analytics are hosted on the N3C Enclave data platform.

The N3C cohort includes COVID-19-positive individuals matched with two COVID-19-negative controls based on up to four sociodemographic variables (age, sex, race, and ethnicity) whenever available in the data partner site. In this analysis, COVID-19 positivity is defined by (1) a set of a priori-defined SARS-CoV-2 laboratory tests (which includes PCR or antigen positivity, but not antibody positivity) or (2) a “strong positive” diagnostic code, with this cohort code being available on GitHub [15,16]. Each data partner site includes all encounters for the cases and controls starting on or after 1 January 2020 with additional encounters from the same data partner site beginning on or after 1 January 2018 (i.e., “lookback data”).

### 2.2. Ethical Reviews

The N3C data transfer to NCATS/NIH is performed under a Johns Hopkins University Reliance Protocol (IRB00249128) or individual site agreements with NIH. Each investigator accessing N3C data received ethics review approvals through a central review at Johns Hopkins University or at the following individual institutions: Medical University of South Carolina, Health Sciences South Carolina Institutional Review Board, Pro00111335, exempt; National Institutes of Health, NIH Office of IRB Operations, exempt; University of Minnesota Institutional Review Board, STUDY00012706, approved; University of Rochester Research Subjects Review Board, STUDY00005366, exempt; University of Washington, Human Subjects Division, STUDY00013147, approved. This work is approved under the Data Use Request [RP-E39D65] N3C Pregnancy Task Team: COVID-19 Incidence, Treatment, and Outcomes in Pregnant Women. The N3C Data Enclave is managed under the authority of the NIH; information can be found at https://ncats.nih.gov/n3c/resources (accessed on 7 December 2023) [17].

### 2.3. Study Design and Analytic Sample 

**Figure 1:**
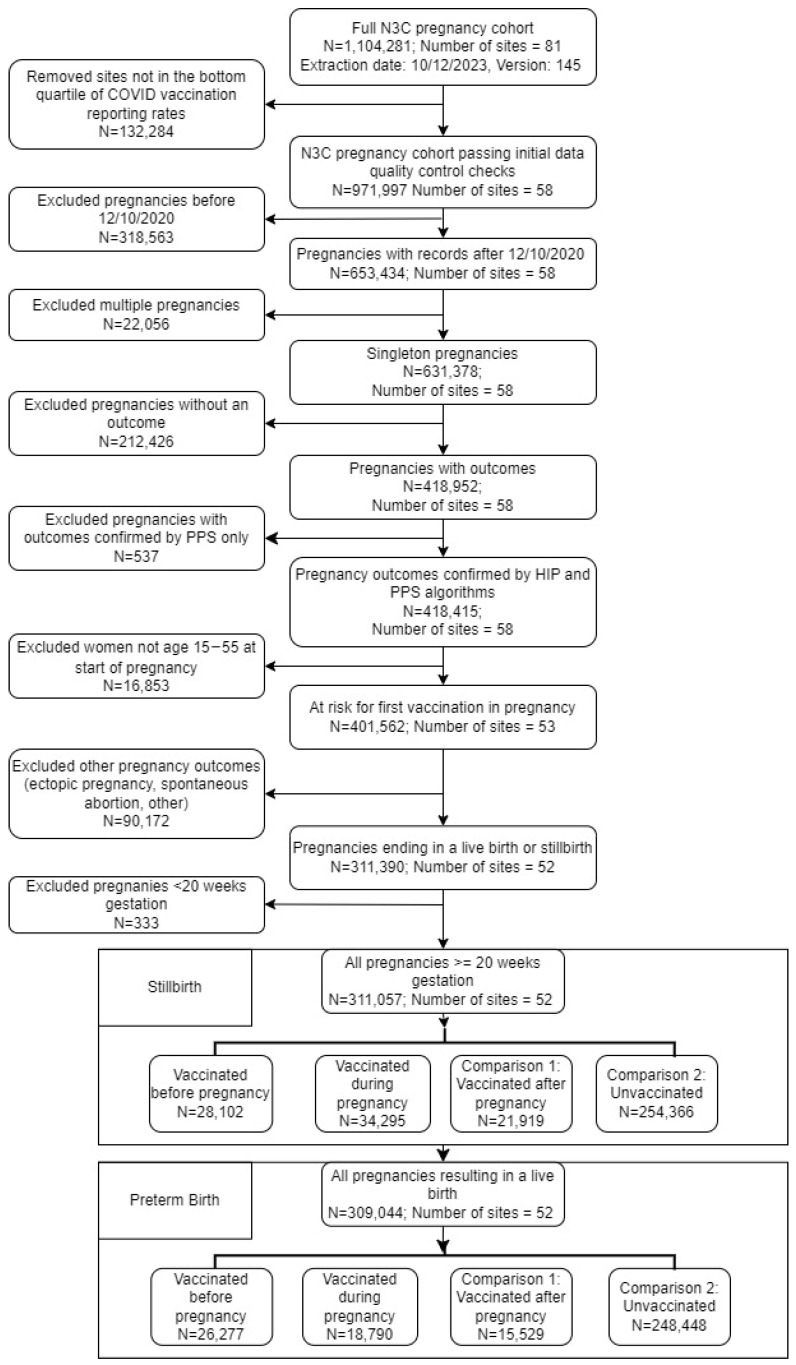
Study inclusion flowchart for pregnant persons included in analysis from U.S. N3C in December 2020–October 2023 period.

The N3C pregnancy domain team previously developed the hierarchy and rule-based pregnancy episode inference integrated with pregnancy progression signatures (HIPPS) approach to identify persons with pregnancy episodes and to estimate gestational age [18]. Our study observation period was from 10 December 2020, when the U.S. Food and Drug Administration (FDA) first authorized a COVID-19 vaccination for general use, to 12 October 2023 (data release version 145) [19]. We included all pregnant persons who had records during the observation period in the N3C Enclave and whose data passed an initial data quality check of removing sites in the bottom quartile of COVID-19 vaccination reporting rates, limiting the data to 58 total contributing sites (Figure 1). Only one pregnancy per person was included. For persons with more than one pregnancy in the study period, the pregnancy episode with the closest date to the first vaccine dose date was used in analysis. For persons without any vaccine, pregnancy episodes were randomly selected within the observation period when more than one pregnancy episode existed for the person using the random function (pyspark.sql.functions.rand), and the first observation in the randomly ordered list was selected. We compared pregnant persons who had a record of first COVID-19 vaccination during pregnancy to two comparison cohorts: (1) pregnant persons who received the first COVID-19 vaccine after pregnancy (Comparison 1) and (2) pregnant persons who had no record of any COVID-19 vaccination during or after the pregnancy episode (Comparison 2). In order to ascertain preterm birth and stillbirth outcomes, we restricted our analyses to pregnant persons who had progressed past 20 weeks of gestational age [20].

### 2.4. Exposures

COVID-19 vaccines approved by the FDA and marketed during our study period were our exposures of interest, including two mRNA vaccines (Pfizer–BioNTech Manufacturing GmbH’s COMIRNATY (Mainz, Germany) and Moderna’s SPIKEVAX (Cambridge, MA, USA)), and a viral vector vaccine (Johnson & Johnson/Janssen’s JCOVDEN (Beerse, Belgium)). For completeness, other vaccines (i.e., AstraZeneca’s VAXZEVRIA (Gaithersburg, MD, USA)) for which exposures were documented in some EHRs and which were aligned with the N3C’s phenotyping rule for COVID-19 vaccination status were not excluded. In the analyses, we focused on adverse outcomes after at least one COVID-19 vaccination of any type before or during pregnancy. 

### 2.5. Outcomes

The two pregnancy outcomes we included were (1) preterm birth and (2) stillbirth; these outcomes were chosen for various reasons, including content knowledge, the existing literature [8,9,21,22], biological plausibility [23], data availability [24], risk profiles of other vaccines [25], our confidence in outcome definition precision in EHR systems [26], and sufficient outcome counts to be powered to estimate associations. Pregnancy outcomes and maternal covariates were defined based on OMOP concept sets and ATLAS (http://atlas-covid19.ohdsi.org/ (accessed on 7 December 2023)), the graphical user interface for the OMOP common data model [24]. The OMOP concept set codes used in this study were curated by obstetric and gynecology specialists and are detailed in Supplementary Table S1. Stillbirth was defined as the outcome if a stillbirth concept appeared in the record at birth or up to two weeks after the pregnancy end date; gestational age was used to differentiate between spontaneous abortion (prior to 20 weeks) or stillbirth (after 20 weeks). We did not include spontaneous abortion as an outcome in this analysis due to the lack of confidence in accurately identifying spontaneous abortions using electronic health records [26]. Additional definition details can also be found in our pregnancy phenotyping publication [18]. Preterm birth was selected as the outcome for pregnancies ending in live births where a preterm concept was noted in the medical record and/or the gestational age was <37 weeks [27]. Preterm birth was disaggregated into extremely preterm at <28 weeks, very preterm from 28 to 32 weeks, and moderate to late preterm from 32 to 37 weeks for descriptive statistics [28]. Due to reporting limitations in identifying the cause of preterm birth in EHR, our analysis does not differentiate between spontaneous and medically induced preterm birth, which may have different clinical implications. Events are enumerated per birth.

### 2.6. Covariates

We included sociodemographic and clinical variables as covariates in our analyses (see Supplementary Tables S1 and S2). Maternal age was a continuous variable based on the age at pregnancy start date. Race/ethnicity included non-Hispanic white, non-Hispanic Black or African American, non-Hispanic American Indian or Alaska Native, non-Hispanic Asian/Asian American, non-Hispanic Native Hawaiian or other Pacific Islander, Hispanic of any race, and other. We classified U.S. residency regions in four categories: Northeast, Midwest, West, or South based on the person’s residential ZIP code. We included insurance type as a categorical variable, including private, public (e.g., Medicare/Medicaid), and other insurance. 

We created an ordinal variable for the number of chronic comorbidities if any of the following pre-existing comorbidities (i.e., prior to the estimated pregnancy start date) were found on each person’s record: type 1 and type 2 diabetes, hypertension, cardiovascular disease, kidney disease, asthma, and obesity. As these conditions are not likely modifiable in a short period, we defined occurrence by the earliest date on N3C. We also constructed a binary variable for smoking or substance use disorder for those who had any record of alcohol, smoking, or substance use disorder. We also included prior experience of preterm birth, stillbirth, or spontaneous abortion as a covariate in each analysis, respectively. 

To account for baseline risk of outcomes and the likelihood of vaccine modifications with the changing predominant variant period, we stratified the data based on the dominant variant period of SARS-CoV-2 (e.g., pre-Delta, Delta, and Omicron) in the U.S. during each person’s pregnancy by assigning each pregnancy to the period with the largest share of gestational days. We also adjusted for COVID-19 infection prior to pregnancy, and if a COVID-19 infection was experienced in the current pregnancy, we adjusted for the trimester in which it occurred. Trimesters were defined as the first (<84 gestational days), second (84–195 gestational days), and third (>195 gestational days) [29].

These covariates were selected based on data availability, completeness, quality, and a priori knowledge of relevance. Missingness for all covariates are included in the descriptive summaries and as its own category in adjusted models.

### 2.7. Statistical Analyses

We present descriptive statistics of demographic and baseline variables outlined above, and we display the gestational age at receipt of first vaccine dose and completion of the vaccine series during pregnancy by week. We conducted Poisson regression for repeated measures using generalized estimating equations (GEEs) to estimate marginal models for the incidence rate of each outcome for each comparison cohort based on their vaccination status during pregnancy, accommodating inherent heterogeneity of outcome ascertainment based on data partner (as a clustering factor, thus accounting for correlations within healthcare system). We did not adjust for the differential duration of time at risk during each period of COVID-19 variant predominance as interest was focused on per-pregnancy episode rates in the following periods: pre-Delta (prior to 20 June 2021), Delta (on or after 20 June 2021 and before 26 December 2021), and Omicron (on or after 26 December 2021) [30]. We present unadjusted incident rate ratios (IRRs) and adjusted IRRs (aIRRs) for each period, focusing on vaccination/timing within pregnancy point estimates and 95% confidence intervals (and Wald *p*-values). As some substrata determined by a unique combination of covariate values may be subject to sparse data (small count of observed outcomes), we restricted adjustments to those mean models consistent with a LASSO-regularized generalized linear model; the regularization parameter value was chosen to be largest within a single Monte Carlo standard error of an optimal value selected via ten-fold cross-validation to yield performant discrimination between those with and without outcomes (i.e., per area under the receiver operating characteristic, or ROC, curve).

Our first analysis estimated the rate of preterm birth among pregnant persons vaccinated during pregnancy compared to pregnant persons vaccinated after pregnancy (Comparison 1), and our second analysis estimated the rate of preterm birth among pregnant persons vaccinated during pregnancy compared to pregnant persons without recorded vaccination, or unvaccinated, in N3C during the analysis period (Comparison 2). Timing of vaccination in gestational weeks is presented by phase of vaccination in Supplementary Figure S1, and frequencies of vaccination by trimester are presented in Table 1. The parallel steps were then followed for stillbirth. In contrast to the preterm birth outcome, stillbirth group comparisons were subject to marked imbalance, a long-recognized issue in modeling count outcomes [31], and small sample cells (e.g., <20 events in some cells) within the various vaccination groups, variant periods, and covariates, which collectively contributed to unstable estimates without special covariate handling (as described in Supplementary Table S3 footnotes). Thus, we chose post hoc analysis to supplement with rate ratios and 95% confidence intervals calculated using exact methods [32]. However, we are currently only able to report the unadjusted estimates from the exact methods, as the N3C Enclave’s computational environment does not support adjusted Poisson regression modeling with exact methods, which is otherwise available with stratification to accommodate data partner heterogeneity in proprietary commercial software (see Methods Supplement) [33]. We are currently exploring ways to optimize our overall approach for adjusted estimates for stillbirth using within-Enclave extensions for exact methods. To adjust for potential confounding due to COVID-19 infection during pregnancy, we ran sensitivity analyses, stratified by documented COVID-19 infection in pregnancy, regardless of trimester, for each model. 

## 3. Results

### 3.1. Sample Description and Baseline Characteristics

A total of 311,057 pregnant people with pregnancies at greater than 20 weeks gestation and 309,044 pregnant people who had pregnancies resulting in live births were included in our analyses (Figure 1). We describe the demographic and clinical characteristics of cases among persons who are at risk for stillbirth and preterm birth in Table 1. Among the pregnant persons, those who were unvaccinated were slightly younger (median age 29, IQR:25-33) compared to those who were vaccinated before (median age: 31, IQR:28-34) or during pregnancy (median age: 31, IQR:27-34) or after pregnancy (median age: 30, IQR:26-34). Black pregnant persons were more commonly vaccinated after pregnancy or unvaccinated (both 19%), white pregnant persons were more commonly vaccinated before (60%) or during pregnancy (57%), and Hispanic pregnant persons were nearly evenly distributed among the different vaccination groups (range of 5–7%). 

The gestational timing of the vaccination based on the final pregnancy outcome is presented in Supplementary Figure S1. Of the vaccinated pregnant persons, the highest percentage was vaccinated during the second trimester (39%), followed by the third trimester (34%) and first trimester (27%) (Table 1). Approximately 78–87% of the persons in our cohort based on vaccination group did not have a COVID-19 infection in their medical records during the current pregnancy. The greatest number of COVID-19 infections were reported during the third trimester, when they are more likely to be detected during screening prior to delivery, ranging from 7 to 10% by vaccination group. The relative distribution of COVID-19 infection timing was similar across vaccination groups. Those who were vaccinated before pregnancy were more likely to have experienced a COVID-19 infection during pregnancy (21.7%) compared to all other groups (13–17%). 

The greatest proportion of persons who were vaccinated during pregnancy (50%) experienced the majority of their pregnancies during the pre-Delta period (prior to 20 June 2021). Similarly, the greatest share of persons who were vaccinated after pregnancy (86%) and unvaccinated persons (46%) experienced the majority of their pregnancies during the pre-Delta period. However, those who were vaccinated before pregnancy experienced most of their pregnancies (73%) during the Omicron variant period (on or after 26 December 2021). Most pregnant persons in each vaccination group did not experience comorbidities (52–55%), followed by one (31–33%) or two (11%) comorbidities. Smoking or substance use was recorded relatively infrequently among all persons (≤1%).

### 3.2. Regression Results for Preterm Birth

**Figure 2:**
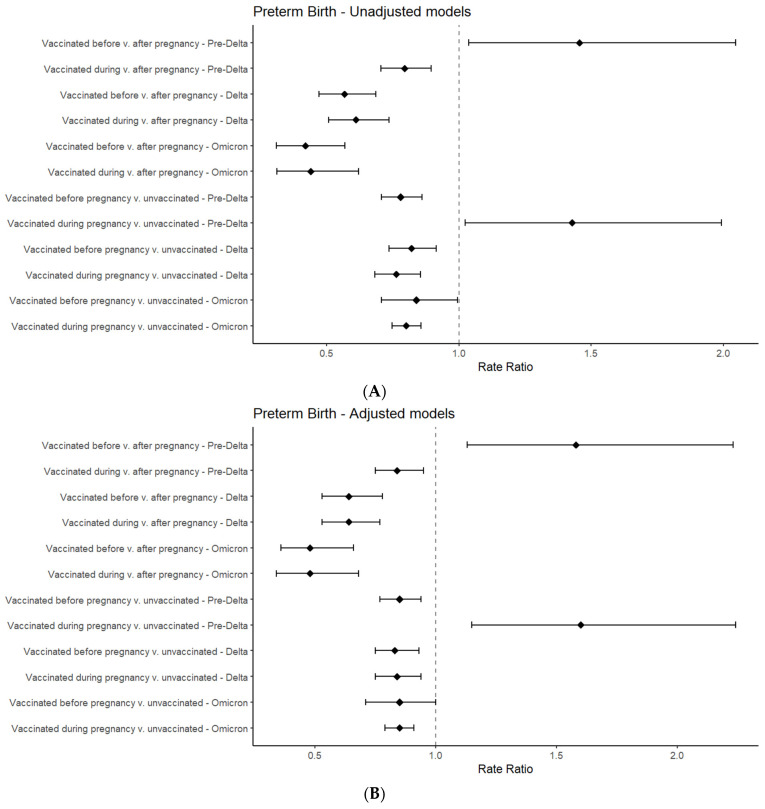
Forest plots for risk ratios of preterm birth based on vaccination status and dominant variant period, generated from unadjusted (**A**) and adjusted (**B**) models *, among pregnant persons in U.S. N3C in December 2020–October 2023 period. * Generated with Poisson regression modeling using robust standard errors (preterm birth) with offset for gestational time in pre-Delta (prior to 20 June 2021), Delta (on or after 20 June 2021 and before 26 December 2021), and Omicron (on or after 26 December 2021) predominant variant periods, and accounting for data partner site.

In this cohort, 17,686 (6%) pregnant persons experienced preterm birth, with 1279 (5%) of pregnant persons vaccinated before pregnancy, 877 (5%) during, and 920 (6%) after pregnancy and with 14,610 (6%) of unvaccinated persons experiencing preterm birth. The aIRRs of preterm birth were generally lower among those who were vaccinated before or during pregnancy in both comparison groups across all variant periods (Figure 2, Supplementary Table S3). In Comparison 1 of the persons who were vaccinated after pregnancy, the aIRR was the lowest for persons who were vaccinated before or during pregnancy who experienced most of their pregnancies during the Omicron predominance period (aIRR: 0.48 for both; 95% CI: 0.36–0.66 before and CI: 0.34–0.68 during pregnancy). In Comparison 1, persons who experienced most of their pregnancies during the pre-Delta variant predominance period had higher aIRRs of preterm birth (vaccinated before pregnancy aIRR 1.58; 95% CI 1.13, 2.23). Similarly, in Comparison 2 of unvaccinated persons, the aIRR was the lowest for those who were vaccinated before and during pregnancy in the Delta variant predominance period (aIRR: 0.83, 95% CI: 0.75–0.93 before pregnancy; aIRR: 0.84, 95% CI: 0.75–0.94 during pregnancy). Persons who experienced most of their pregnancies during the pre-Delta variant predominance period had a greater aIRR of preterm birth (vaccinated during pregnancy aIRR: 1.60; 95% CI: 1.15, 2.24) compared to unvaccinated persons. The sensitivity analyses stratified by COVID-19 infection during pregnancy showed that persons who were vaccinated before and during pregnancy, regardless of their history of COVID-19 infection, had a lower aIRR of preterm birth than those who were vaccinated after pregnancy or those who were not vaccinated (Supplementary Table S3). The one exception was among two comparisons with the majority of pregnancy durations in the pre-Delta variance predominance period, where not having a COVID-19 infection and being vaccinated before or during pregnancy was associated with a higher risk of preterm birth. 

### 3.3. Regression Results for Stillbirth

**Figure 3:**
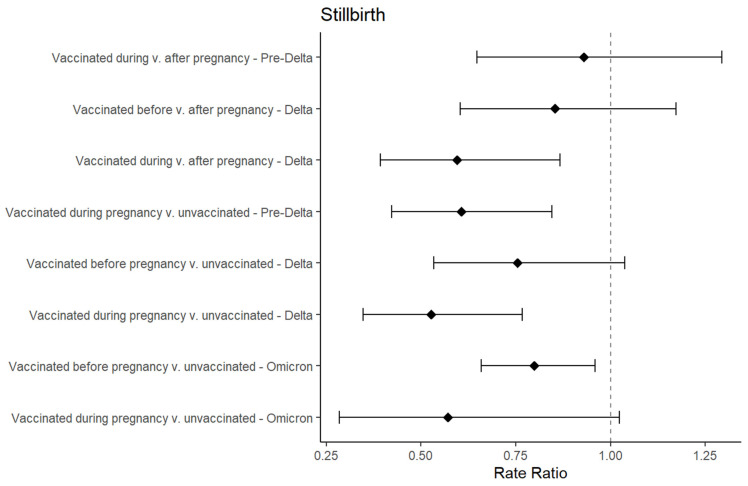
Forest plot for risk ratios of stillbirths based on vaccination status and dominant variant period, generated from unadjusted models *, among pregnant persons in U.S. N3C in December 2020–October 2023 period. * Generated with unadjusted exact regression modeling using robust standard errors with offset for gestational time in pre-Delta (prior to 20 June 2021), Delta (on or after 20 June 2021 and before 26 December 2021), and Omicron (on or after 26 December 2021) predominant variant periods. We did not account for data partner sites or covariates in adjusted model due to current analytic limitations in N3C Enclave. Not all comparison groups for stillbirth are presented due to small sample sizes (<20 in cells of each comparison group).

A total of 2013 pregnant persons experienced stillbirths; 156 persons (0.6%) vaccinated before pregnancy, 73 persons (0.4%) vaccinated during pregnancy, 68 persons (0.4%) vaccinated after pregnancy, and 1716 (0.7%) unvaccinated pregnant persons experienced stillbirths. Comparisons where cell counts were <20 were omitted. In Comparison 1 of those who were vaccinated after pregnancy, the IRR was the lowest for those with pregnancies in the Delta period who were vaccinated during pregnancy (IRR: 0.60, 95% CI: 0.39–0.87) and the highest among persons with pregnancies during the pre-Delta variant predominance period who were vaccinated during pregnancy (IRR: 1.82, 95% CI: 0.37–5.35) (Figure 3). For Comparison 2 of unvaccinated pregnant persons, the IRR was the lowest among those with pregnancies in the Delta variant predominance period who were vaccinated during pregnancy (IRR: 0.53, 95% CI: 0.35–0.77) and the highest among persons with pregnancies during the Pre-Delta variant predominance period who were vaccinated before pregnancy (1.19, 95% CI: 0.24–3.49). The sensitivity analyses stratified by COVID-19 infection during pregnancy showed that persons who were vaccinated before and during pregnancy, regardless of their history of COVID-19 infection, did not have a higher risk of stillbirth compared to those who were vaccinated after pregnancy or unvaccinated people.

## 4. Discussion

This analysis of preterm birth and stillbirth outcomes after COVID-19 vaccination adds to the body of evidence supporting the safety of the COVID-19 vaccination among pregnant persons. We largely did not find adverse associations between vaccination and these pregnancy outcomes after controlling for the dominant variant period in each pregnancy and other potential confounders. Interestingly, at times, we observed a protective effect of COVID-19 vaccination against preterm birth between both groups of pregnant persons who were vaccinated before or during pregnancy against both comparator groups of those who were vaccinated after pregnancy and unvaccinated pregnant persons. Overall, our findings corroborate an existing body of evidence [34,35], which is reassuring for the safety of COVID-19 vaccination during pregnancy. Our specific findings should bolster confidence for pregnant persons, providers, and policymakers by continuing to highlight the importance of COVID-19 vaccination for this group despite the end of the public health emergency. 

While the acute phase of the COVID-19 emergency has ended [36], COVID-19 vaccination remains important, especially for the most vulnerable groups who may be at greater risk for more severe disease, such as pregnant persons or the immunologically naive population of newborns. Additionally, the confluence of respiratory syncytial virus (RSV) disease, influenza, and COVID-19 during “respiratory virus season” may impact pregnant persons and their fetuses. Studies of COVID-19 vaccination in pregnancy have demonstrated effectiveness against incident COVID-19 infection and severity for both pregnant persons and their infants [37]. Thus, with high effectiveness data coupled with a reassuring safety profile of COVID-19 vaccination, these findings collectively support vaccination in pregnancy to prevent severe health outcomes for pregnant persons and infants. Additionally, numerous studies show that COVID-19 infections during pregnancy can result in similar respiratory and other adverse outcomes for both pregnant persons and infants (e.g., preterm birth, stillbirth, hospitalizations, and even infant neurodevelopmental delays) [38,39]. In the case of preterm birth, we found a potential trend of a protective effect from COVID-19 vaccination. This trend may be due, however, to additional or residual confounding, e.g., positive healthcare-seeking behavior as evidenced by receipt of a COVID-19 vaccine, or due to the reduced risk of COVID-19 infection during pregnancy positively impacting pregnancy outcomes and reducing the risk of preterm birth, as suggested by a recent ecologic study [40]. Our models stratified by COVID-19 infection during pregnancy did not necessarily corroborate a higher risk of preterm birth among persons who also had COVID-19 infection documented during pregnancy; these findings should be interpreted with caution as a sparsity of data across the various comparator groups could limit inferences, especially for stillbirth. Concerns about the misclassification of COVID-19 vaccination could also be more influential for comparisons of smaller sample sizes. Future work should more carefully examine the mediation or moderation of this risk by COVID-19 infection as well as vaccination.

The collective evidence for mRNA COVID-19 vaccine safety and effectiveness is promising in terms of mRNA technologies for other diseases or conditions. Our findings may also support the possibility of using mRNA vaccines for other illnesses in pregnant persons, as others have suggested [41]. The success of the COVID-19 mRNA vaccine was a catalyst for bringing the RSV vaccine ABRYSVO to market in 2023, and mRNA vaccines against other respiratory viruses are under development, including those for avian influenza and parainfluenza [42]. Self-amplifying RNA vaccines, a modification of mRNA technology, have demonstrated potential for other disease targets in preclinical and phase 1 trials, including for rabies, HIV, and some cancers [43]. Existing safety data, including but not limited to the results of this study, may contribute to the approval of future mRNA vaccines for pregnant persons in these other diseases and conditions of which pregnant persons are especially vulnerable. Further advocacy through anticipatory consensus statements or society guidelines from vaccination, immunology, or pregnancy groups could advance the efficient use of future mRNA-based technologies for pregnant persons. 

Given the overall real-world safety and effectiveness evidence for most drugs, including vaccines, there are also calls for a greater inclusion of pregnant persons in trials for drugs and biologics. Over the past two decades, a paradigm shift has been occurring in the ethics of risk and benefit to pregnant persons, fetuses, and infants. Most drugs that pregnant persons may use for varied acute or chronic conditions have not had explicit safety and efficacy testing in pregnant persons during the approval processes, resulting in evidence gaps about the appropriate dosing and timing during pregnancy [44]. There is an ethical argument that the lack of inclusion of pregnant persons and persons at risk of pregnancy in some trials, such as that which has been made in HIV treatment and prevention trials, has caused overall harm to these persons who have not been able to readily benefit from the approved drugs [45,46,47]. Similarly, there is a movement in bioethics to frame pregnant persons as a “complex” or “special” population—with special considerations of the immunological status of pregnancy and risks to the pregnant person and fetus—rather than excluding them from studies due to being in the historical “vulnerable populations” category [45]. Perhaps the lessons learned from this study and other safety analyses of COVID-19 vaccination may help engender confidence in this more inclusive movement of greater trial or study inclusivity of pregnant persons for future emerging infections. 

### Strengths and Limitations

Our work has several strengths that include the following: (1) a large, nationally sampled dataset with large numbers of rare outcomes that allowed us to detect statistically significant associations, (2) our selection of pregnancy outcomes of preterm birth and stillbirth that are traditionally well captured in EHR and are clinically meaningful, (3) our ability to control for many possible confounders, including the changing risk over the various variant periods, and (4) a highly vetted phenotype for COVID-19 vaccination and a validated pregnancy cohort [18,48]. However, there are several limitations to our work as well: (1) We anticipate some misclassification for the outcomes, exposures, and covariates in our model. The frequency of preterm birth may be underestimated, as 5–6% of persons in each vaccination group experienced preterm births, which is lower than the national frequency of approximately 10% of live births in the U.S. Similarly, vaccination status in N3C may be misclassified, as we group all patients without documented vaccination as unvaccinated, which may not capture vaccines that were received in outpatient settings, and the overall vaccination rates are lower in N3C compared to those reported by CDC [49]. Though incident COVID-19 infections may also be underreported in EHR, after the wide-spread use of home antigen-based testing, we anticipate that this misclassification will not be large among pregnant persons undergoing universal testing for hospital admissions for deliveries. However, we anticipate this misclassification to be non-differential across the various vaccination groups and variant periods; thus, our estimates would be biased towards the null. Other important potential confounders may be underrepresented (i.e., smoking, <1% of all pregnant persons) or difficult to distinguish in medical records (other health seeking behaviors). (2) We were unable to adjust for or conduct secondary analyses based on some confounders, such as primigravida status, due to the small sample size of the outcomes, especially for stillbirths. (3) Many data site partners participating in N3C include academic health centers, which may limit their generalizability to other health systems; however, this may result in the inclusion of more complicated pregnancies and births through referral and is not likely to underestimate the occurrence of outcomes. Our overall rates of preterm births and stillbirths were consistent with the rates in the general U.S. population [50,51].

## 5. Conclusions

This study reinforces the safety of vaccination for COVID-19 in pregnant persons in terms of the potential adverse outcomes of preterm births and stillbirths in the implementation setting. In some variant periods, the preterm birth rates were lower among persons who were vaccinated before or during pregnancy, suggesting a protective effect. No consistent trends were associated with a higher risk with vaccination. This study affirms evidence for the safety of COVID-19 vaccines, including mRNA vaccines, over time among pregnant persons, engendering confidence for pregnant persons, providers, and policymakers in continuing to highlight the importance of COVID-19 vaccination for this group.

## Figures and Tables

**Table 1:** Baseline characteristics of pregnant persons included in analysis ^1^ by timing of vaccination among pregnant persons in U.S. N3C in December 2020–October 2023 period.

Variable Category	Category Level	Vaccinated before Pregnancy n = 26,433	Vaccinated during Pregnancy n = 18,863	Vaccinated after Pregnancy n = 15,597	Unvaccinated n = 250,164
Age in years, median (IQR)		31 (28–34)	31 (27–34)	30 (26–34)	29 (25–33)
Race/ethnicity ^2^	American Indian or Alaska Native	178 (0.7%)	72 (0.4%)	75 (0.5%)	1073 (0.4%)
	Asian/Asian American	1667 (6.3%)	1173 (6.2%)	917 (5.9%)	11,019 (4.4%)
	Black/African American	3156 (11.9%)	2548 (13.5%)	2883 (18.5%)	47,893 (19.1%)
	Hawaiian or Pacific Islander	157 (0.6%)	79 (0.4%)	68 (0.4%)	1180 (0.5%)
	Hispanic	1437 (5.4%)	1136 (6%)	978 (6.3%)	17,139 (6.9%)
	Other	168 (0.6%)	81 (0.4%)	104 (0.7%)	2182 (0.9%)
	White	15,793 (59.7%)	10,648 (56.4%)	7897 (50.6%)	128,674 (51.4%)
	Missing	3877 (14.7%)	3126 (16.6%)	2675 (17.2%)	41,004 (16.4%)
Region of participant residence	Midwest	3295 (12.5%)	2696 (14.3%)	1799 (11.5%)	36,194 (14.5%)
	Northeast	1596 (6%)	1986 (10.5%)	1841 (11.8%)	23,425 (9.4%)
	South	10,627 (40.2%)	7338 (38.9%)	5370 (34.4%)	61,819 (24.7%)
	West	3947 (14.9%)	2329 (12.3%)	1777 (11.4%)	22,479 (9%)
	Missing	6968 (26.4%)	4514 (23.9%)	4810 (30.8%)	10,6247 (42.5%)
Insurance	Medicare/Medicaid	2148 (8.1%)	2019 (10.7%)	1829 (11.7%)	35,433 (14.2%)
	Private	6570 (24.9%)	4703 (24.9%)	3414 (21.9%)	38,763 (15.5%)
	Other	1127 (4.3%)	592 (3.1%)	434 (2.8%)	2480 (1%)
	None/Missing	16,588 (62.8%)	11,549 (61.2%)	9920 (63.6%)	173,488 (69.3%)
Dominant COVID-19 variant period for majority of pregnancy duration	Pre-Delta	410 (1.6%)	9357 (49.6%)	13,425 (86.1%)	115,043 (46%)
	Delta	6814 (25.8%)	6936 (36.8%)	1836 (11.8%)	54,806 (21.9%)
	Omicron	19,209 (72.7%)	2570 (13.6%)	336 (2.2%)	80,315 (32.1%)
Primigravida		2446 (9.3%)	1803 (9.6%)	1298 (8.3%)	19,121 (7.6%)
Number of chronic comorbidities	None	13,735 (52%)	10,276 (54.5%)	8178 (52.4%)	129,128 (51.6%)
	1	8354 (31.6%)	5764 (30.6%)	5021 (32.2%)	82,359 (32.9%)
	2	3015 (11.4%)	1974 (10.5%)	1685 (10.8%)	27,810 (11.1%)
	3	907 (3.4%)	608 (3.2%)	524 (3.4%)	7916 (3.2%)
	4	308 (1.2%)	182 (1%)	134 (0.9%)	2181 (0.9%)
	5 or more	114 (0.4%)	59 (0.3%)	55 (0.4%)	770 (0.3%)
Chronic comorbidities	Hypertension	2204 (8.3%)	1312 (7%)	1080 (6.9%)	16,071 (6.4%)
	Kidney disease	564 (2.1%)	369 (2%)	284 (1.8%)	4123 (1.6%)
	Type 1 diabetes	333 (1.3%)	252 (1.3%)	188 (1.2%)	2818 (1.1%)
	Type 2 diabetes	1046 (4%)	708 (3.8%)	538 (3.4%)	8664 (3.5%)
	Asthma	3907 (14.8%)	2569 (13.6%)	2213 (14.2%)	34,501 (13.8%)
	Cardiovascular disease	2711 (10.3%)	1718 (9.1%)	1381 (8.9%)	25,937 (10.4%)
	Chronic obstructive pulmonary disease	58 (0.2%)	35 (0.2%)	37 (0.2%)	508 (0.2%)
	Obesity	8117 (30.7%)	5613 (29.8%)	5069 (32.5%)	81,907 (32.7%)
Any smoking or substance use		270 (1%)	<20	<20	3126 (1.2%)
Any prior stillbirth		114 (0.4%)	94 (0.5%)	55 (0.4%)	1265 (0.5%)
Any prior preterm birth		598 (2.3%)	328 (1.7%)	267 (1.7%)	5281 (2.1%)
Trimester of COVID-19 infection in current pregnancy	No COVID-19 infection recorded during pregnancy	20,696 (78.3%)	16,203 (85.9%)	13,545 (86.8%)	206,657 (82.6%)
	1st trimester	1077 (4.1%)	437 (2.3%)	341 (2.2%)	5660 (2.3%)
	2nd trimester	2035 (7.7%)	818 (4.3%)	609 (3.9%)	12,778 (5.1%)
	3rd trimester	2625 (9.9%)	1405 (7.4%)	1102 (7.1%)	25,069 (10%)
Trimester of COVID-19 vaccination in current pregnancy	1st trimester	-	5047 (27%)	-	-
	2nd trimester	-	7365 (39%)	-	-
	3rd trimester	-	6451 (34%)	-	-
Experienced COVID-19 infection prior to pregnancy		4194 (15.9%)	1120 (5.9%)	414 (2.7%)	15,327 (6.1%)
Outcome of current pregnancy: preterm birth		1279 (4.8%)	877 (4.6%)	920 (5.9%)	14,610 (5.8%)
Outcome of current pregnancy: stillbirth		156 (0.6%)	73 (0.4%)	68 (0.4%)	1716 (0.7%)
Manufacturer of first COVID-19 vaccine received	AstraZeneca	<20	-	-	-
	Janssen	1466 (5.5%)	688 (3.6%)	760 (4.9%)	-
	Moderna	6410 (24.2%)	3703 (19.6%)	3033 (19.4%)	-
	Pfizer	18,171 (68.7%)	14,011 (74.3%)	11,515 (73.8%)	-
	Missing	369 (1.4%)	461 (2.4%)	289 (1.9%)	-

^1^ Note: The preterm birth analysis cohort was further restricted to a total sample of 309,044 persons who experienced live births, including 26,277 who were vaccinated before, 18,790 vaccinated during, and 15,529 vaccinated after pregnancy and 248,448 unvaccinated persons. ^2^ All race/ethnicity categories labeled without “Hispanic” are non-Hispanic, and the Hispanic category can be any race with that ethnicity.

## Data Availability

The N3C data transfer to NCATS was performed under a Johns Hopkins University Reliance Protocol # IRB00249128 or individual site agreements with NIH. The N3C Data Enclave is managed under the authority of the NIH; information can be found at ncats.nih.gov/n3c/resources (accessed on 7 December 2023). Enclave data are protected and can be accessed for COVID-19-related research with an NIH-approved (1) IRB protocol and (2) institutional Data Use Request (DUR). A detailed account of data protections and access tiers can be found at https://ncats.nih.gov/n3c/resources/data-access (accessed on 7 December 2023). Enclave and data access instructions can be found at https://covid.cd2h.org/for-researchers (accessed on 7 December 2023); all codes used to produce the analyses in this manuscript are available within the N3C Enclave to users with valid login credentials to support reproducibility.

## Supplementary Materials

The following supporting information can be downloaded at https://www.mdpi.com/article/10.3390/vaccines12030289/s1, Methods Supplement. Table S1: Concept sets of covariates. Table S2: Covariate and outcome definitions. Table S3: Unadjusted and adjusted incidence rate ratios (aIRR) of preterm birth based on vaccination status and dominant variation period among pregnant persons in U.S. N3C in December 2020–October 2023 period. Table S4: Unadjusted incidence rate ratios of stillbirth based on vaccination status and dominant variation period among pregnant persons in U.S. N3C in December 2020–October 2023 period. Table S5: Sensitivity analyses stratified by COVID-19 infection during pregnancy. Figure S1: Density plot of gestational weeks and timing of vaccination.

## N3C Attribution

The analyses described in this publication were conducted with data or tools accessed through the NCATS N3C Data Enclave https://covid.cd2h.org (accessed on 7 December 2023) and N3C Attribution & Publication Policy v 1.2-2020-08-25b supported by NCATS U24 TR002306, Axle Informatics Subcontract: NCATS-P00438-B. This research was possible because of the patients whose information is included within the data and the organizations (https://ncats.nih.gov/n3c/resources/data-contribution/data-transfer-agreement-signatories (accessed on 7 December 2023)) and scientists who have contributed to the ongoing development of this community resource [15].

## Data Partners with Released Data

The following institutions whose data are released or pending: Available: Advocate Health Care Network—UL1TR002389: The Institute for Translational Medicine (ITM) • Aurora Health Care Inc—UL1TR002373: Wisconsin Network For Health Research • Boston University Medical Campus—UL1TR001430: Boston University Clinical and Translational Science Institute • Brown University—U54GM115677: Advance Clinical Translational Research (Advance-CTR) • Carilion Clinic—UL1TR003015: iTHRIV Integrated Translational health Research Institute of Virginia • Case Western Reserve University—UL1TR002548: The Clinical & Translational Science Collaborative of Cleveland (CTSC) • Charleston Area Medical Center—U54GM104942: West Virginia Clinical and Translational Science Institute (WVCTSI) • Children’s Hospital Colorado—UL1TR002535: Colorado Clinical and Translational Sciences Institute • Columbia University Irving Medical Center—UL1TR001873: Irving Institute for Clinical and Translational Research • Dartmouth College—None (Voluntary) Duke University—UL1TR002553: Duke Clinical and Translational Science Institute • George Washington Children’s Research Institute—UL1TR001876: Clinical and Translational Science Institute at Children’s National (CTSA-CN) • George Washington University—UL1TR001876: Clinical and Translational Science Institute at Children’s National (CTSA-CN) • Harvard Medical School—UL1TR002541: Harvard Catalyst • Indiana University School of Medicine—UL1TR002529: Indiana Clinical and Translational Science Institute • Johns Hopkins University—UL1TR003098: Johns Hopkins Institute for Clinical and Translational Research • Louisiana Public Health Institute—None (Voluntary) • Loyola Medicine—Loyola University Medical Center • Loyola University Medical Center—UL1TR002389: The Institute for Translational Medicine (ITM) • Maine Medical Center—U54GM115516: Northern New England Clinical & Translational Research (NNE-CTR) Network • Mary Hitchcock Memorial Hospital & Dartmouth Hitchcock Clinic—None (Voluntary) • Massachusetts General Brigham—UL1TR002541: Harvard Catalyst • Mayo Clinic Rochester—UL1TR002377: Mayo Clinic Center for Clinical and Translational Science (CCaTS) • Medical University of South Carolina—UL1TR001450: South Carolina Clinical & Translational Research Institute (SCTR) • MITRE Corporation—None (Voluntary) • Montefiore Medical Center—UL1TR002556: Institute for Clinical and Translational Research at Einstein and Montefiore • Nemours—U54GM104941: Delaware CTR ACCEL Program • NorthShore University HealthSystem—UL1TR002389: The Institute for Translational Medicine (ITM) • Northwestern University at Chicago—UL1TR001422: Northwestern University Clinical and Translational Science Institute (NUCATS) • OCHIN—INV-018455: Bill and Melinda Gates Foundation grant to Sage Bionetworks • Oregon Health & Science University—UL1TR002369: Oregon Clinical and Translational Research Institute • Penn State Health Milton S. Hershey Medical Center—UL1TR002014: Penn State Clinical and Translational Science Institute • Rush University Medical Center—UL1TR002389: The Institute for Translational Medicine (ITM) • Rutgers, The State University of New Jersey—UL1TR003017: New Jersey Alliance for Clinical and Translational Science • Stony Brook University—U24TR002306 • The Alliance at the University of Puerto Rico, Medical Sciences Campus—U54GM133807: Hispanic Alliance for Clinical and Translational Research (The Alliance) • The Ohio State University—UL1TR002733: Center for Clinical and Translational Science • The State University of New York at Buffalo—UL1TR001412: Clinical and Translational Science Institute • The University of Chicago—UL1TR002389: The Institute for Translational Medicine (ITM) • The University of Iowa—UL1TR002537: Institute for Clinical and Translational Science • The University of Miami Leonard M. Miller School of Medicine—UL1TR002736: University of Miami Clinical and Translational Science Institute • The University of Michigan at Ann Arbor—UL1TR002240: Michigan Institute for Clinical and Health Research • The University of Texas Health Science Center at Houston—UL1TR003167: Center for Clinical and Translational Sciences (CCTS) • The University of Texas Medical Branch at Galveston—UL1TR001439: The Institute for Translational Sciences • The University of Utah—UL1TR002538: Uhealth Center for Clinical and Translational Science • Tufts Medical Center—UL1TR002544: Tufts Clinical and Translational Science Institute • Tulane University—UL1TR003096: Center for Clinical and Translational Science • The Queens Medical Center—None (Voluntary) • University Medical Center New Orleans—U54GM104940: Louisiana Clinical and Translational Science (LA CaTS) Center • University of Alabama at Birmingham—UL1TR003096: Center for Clinical and Translational Science • University of Arkansas for Medical Sciences—UL1TR003107: UAMS Translational Research Institute • University of Cincinnati—UL1TR001425: Center for Clinical and Translational Science and Training • University of Colorado Denver, Anschutz Medical Campus—UL1TR002535: Colorado Clinical and Translational Sciences Institute • University of Illinois at Chicago—UL1TR002003: UIC Center for Clinical and Translational Science • University of Kansas Medical Center—UL1TR002366: Frontiers: University of Kansas Clinical and Translational Science Institute • University of Kentucky—UL1TR001998: UK Center for Clinical and Translational Science • University of Massachusetts Medical School Worcester—UL1TR001453: The UMass Center for Clinical and Translational Science (UMCCTS) • University Medical Center of Southern Nevada—None (voluntary) • University of Minnesota—UL1TR002494: Clinical and Translational Science Institute • University of Mississippi Medical Center—U54GM115428: Mississippi Center for Clinical and Translational Research (CCTR) • University of Nebraska Medical Center—U54GM115458: Great Plains IDeA-Clinical & Translational Research • University of North Carolina at Chapel Hill—UL1TR002489: North Carolina Translational and Clinical Science Institute • University of Oklahoma Health Sciences Center—U54GM104938: Oklahoma Clinical and Translational Science Institute (OCTSI) • University of Pittsburgh—UL1TR001857: The Clinical and Translational Science Institute (CTSI) • University of Pennsylvania—UL1TR001878: Institute for Translational Medicine and Therapeutics • University of Rochester—UL1TR002001: UR Clinical & Translational Science Institute • University of Southern California—UL1TR001855: The Southern California Clinical and Translational Science Institute (SC CTSI) • University of Vermont—U54GM115516: Northern New England Clinical & Translational Research (NNE-CTR) Network • University of Virginia—UL1TR003015: iTHRIV Integrated Translational health Research Institute of Virginia • University of Washington—UL1TR002319: Institute of Translational Health Sciences • University of Wisconsin-Madison—UL1TR002373: UW Institute for Clinical and Translational Research • Vanderbilt University Medical Center—UL1TR002243: Vanderbilt Institute for Clinical and Translational Research • Virginia Commonwealth University—UL1TR002649: C. Kenneth and Dianne Wright Center for Clinical and Translational Research • Wake Forest University Health Sciences—UL1TR001420: Wake Forest Clinical and Translational Science Institute • Washington University in St. Louis—UL1TR002345: Institute of Clinical and Translational Sciences • Weill Medical College of Cornell University—UL1TR002384: Weill Cornell Medicine Clinical and Translational Science Center • West Virginia University—U54GM104942: West Virginia Clinical and Translational Science Institute (WVCTSI) Submitted: Icahn School of Medicine at Mount Sinai—UL1TR001433: ConduITS Institute for Translational Sciences • The University of Texas Health Science Center at Tyler—UL1TR003167: Center for Clinical and Translational Sciences (CCTS) • University of California, Davis—UL1TR001860: UCDavis Health Clinical and Translational Science Center • University of California, Irvine—UL1TR001414: The UC Irvine Institute for Clinical and Translational Science (ICTS) • University of California, Los Angeles—UL1TR001881: UCLA Clinical Translational Science Institute • University of California, San Diego—UL1TR001442: Altman Clinical and Translational Research Institute • University of California, San Francisco—UL1TR001872: UCSF Clinical and Translational Science Institute Pending: Arkansas Children’s Hospital—UL1TR003107: UAMS Translational Research Institute • Baylor College of Medicine—None (Voluntary) • Children’s Hospital of Philadelphia—UL1TR001878: Institute for Translational Medicine and Therapeutics • Cincinnati Children’s Hospital Medical Center—UL1TR001425: Center for Clinical and Translational Science and Training • Emory University—UL1TR002378: Georgia Clinical and Translational Science Alliance • HonorHealth—None (Voluntary) • Loyola University Chicago—UL1TR002389: The Institute for Translational Medicine (ITM) • Medical College of Wisconsin—UL1TR001436: Clinical and Translational Science Institute of Southeast Wisconsin • MedStar Health Research Institute—None (Voluntary) • Georgetown University—UL1TR001409: The Georgetown-Howard Universities Center for Clinical and Translational Science (GHUCCTS) • MetroHealth—None (Voluntary) • Montana State University—U54GM115371: American Indian/Alaska Native CTR • NYU Langone Medical Center—UL1TR001445: Langone Health’s Clinical and Translational Science Institute • Ochsner Medical Center—U54GM104940: Louisiana Clinical and Translational Science (LA CaTS) Center • Regenstrief Institute—UL1TR002529: Indiana Clinical and Translational Science Institute • Sanford Research—None (Voluntary) • Stanford University—UL1TR003142: Spectrum: The Stanford Center for Clinical and Translational Research and Education • The Rockefeller University—UL1TR001866: Center for Clinical and Translational Science • The Scripps Research Institute—UL1TR002550: Scripps Research Translational Institute • University of Florida—UL1TR001427: UF Clinical and Translational Science Institute • University of New Mexico Health Sciences Center—UL1TR001449: University of New Mexico Clinical and Translational Science Center • University of Texas Health Science Center at San Antonio—UL1TR002645: Institute for Integration of Medicine and Science • Yale New Haven Hospital—UL1TR001863: Yale Center for Clinical Investigation.
