# Supplementary material for: Pregnancy Outcomes among Pregnant Persons after COVID-19 Vaccination: Assessing Vaccine Safety in Retrospective Cohort Analysis of U.S. National COVID Cohort Collaborative (N3C)
Source: Vaccines (Basel). 2024 Mar 11;12(3):289. doi: 10.3390/vaccines12030289 (PMC10975285; doi:10.3390/vaccines12030289)
Supplement: Supplementary file 1 [file vaccines-12-00289-s001.zip › Methods Supplement.pdf]

Methods Supplement

In contrast to the preterm birth outcome, stillbirth group comparisons were subject to marked imbalance, a long-recognized issue in modeling count outcomes (see, for example, section 9.1 of Hilbe 2014) and small sample cells. A further complication arose when this relatively rare outcome of stillbirth gets cross-classified in terms of multiple categorical adjustment variables, requiring use of exact methods whose nominal significance levels are maintained by enumerating permutations conditional on sufficient statistics consistent with the observed data.[44] Altogether, once noting that small sample cells (e.g., <20 events in some cells) within the various vaccination groups, and by variant periods, and covariates generated unstable estimates. Thus, we chose *post-hoc* to not report the large-sample-approximation-based estimates generated from small sample cells, while caveating those concurrently estimated via generalized estimating equations (GEEs) that account for data partner heterogeneity. Currently, the N3C Enclave's computational environment does not support adjusted (Poisson regression) modeling with exact methods as available in proprietary commercial software[26] but not yet implemented in open-source R/Python packages to the best of our knowledge; we are currently exploring ways to optimize our overall approach for inference in imbalanced/sparse-cell-count instances using within-Enclave extensions to alternative binary-outcome models with exact methods.

We selected a continuous measure of age to adjust for any potential differential outcome risk by age yet to preserve both interpretability that is most generalizable as well as degrees of freedom spent within models, given sample size limitations within each covariate level (i.e., each unique combination of other adjusting covariates). To further confirm the tenability of linear-in-age-at-index-date (e.g., pregnancy-start), we (i) assessed percent change in a measure helpful for model selection when fitting via GEEs, the quasi-likelihood information criterion (QIC) and changes from adding additional nonlinear (quadratic or cubic) terms for continuous age; and (ii) explored smooths of the final models' respective residuals versus continuous age, inspecting each for markedly nonlinear trends. Both approaches confirmed that each model adjusting for all other

covariates along with linear continuous-age terms did not exhibit (<1% change in QIC and loess smooths of residuals consistent with horizontal lines).
