# Supplementary material for: Pregnancy Outcomes among Pregnant Persons after COVID-19 Vaccination: Assessing Vaccine Safety in Retrospective Cohort Analysis of U.S. National COVID Cohort Collaborative (N3C)
Source: Vaccines (Basel). 2024 Mar 11;12(3):289. doi: 10.3390/vaccines12030289 (PMC10975285; doi:10.3390/vaccines12030289)
Supplement: Supplementary file 1 [file vaccines-12-00289-s001.zip › Supplementary Figure S1.pdf]

Figure S1: Density plot of gestational weeks and timing of vaccination

1A.

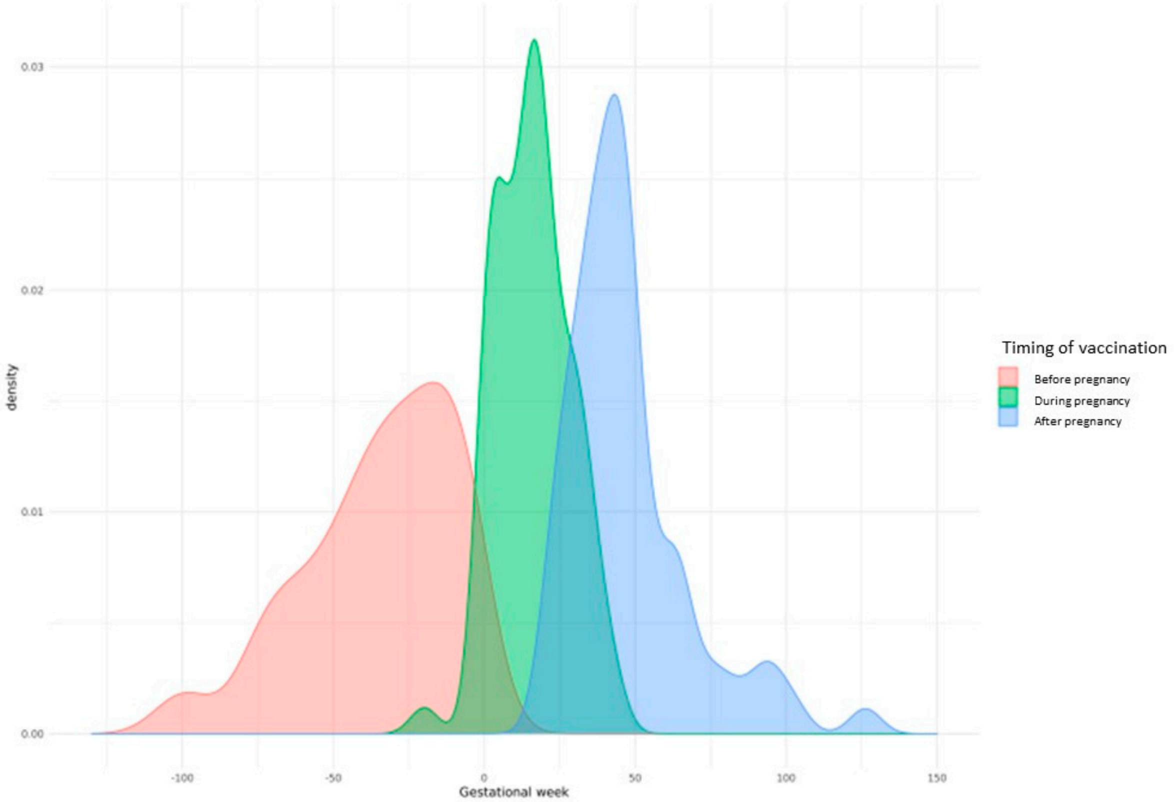

1B.

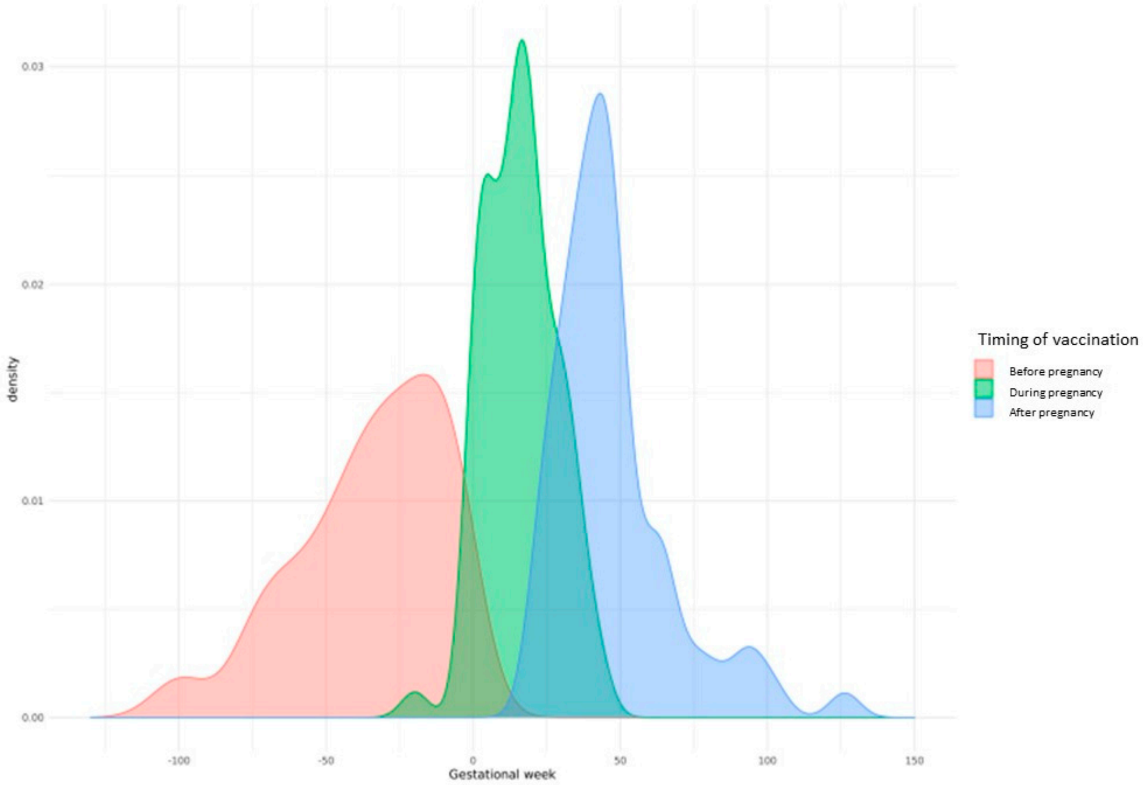

Figure S1. Density plot of weeks in relation to the pregnancy (gestational age by week)* and first vaccine dose receipt for persons experiencing preterm birth (2A) and stillbirth (2B) among pregnant persons in U.S. N3C, December 2020-October 2023. * Note: The x-axis is presented in weeks in relation to the pregnancy, thus labeled "gestational week," where gestational week = 0 is the estimated date of conception. Time prior to gestational week = 0 indicates time before pregnancy.
