# Supplementary material for: Pregnancy Outcomes among Pregnant Persons after COVID-19 Vaccination: Assessing Vaccine Safety in Retrospective Cohort Analysis of U.S. National COVID Cohort Collaborative (N3C)
Source: Vaccines (Basel). 2024 Mar 11;12(3):289. doi: 10.3390/vaccines12030289 (PMC10975285; doi:10.3390/vaccines12030289)
Supplement: Supplementary file 1 [file vaccines-12-00289-s001.zip › Table S3. Unadjusted and adjusted incidence rate ratios (aIRR) of preterm birth by vaccination status and dominant variation period.pdf]

Table S3. Unadjusted and adjusted incidence rate ratios (aIRR) of preterm birth by vaccination status and dominant variation period among pregnant persons in the U.S. N3C, December 2020-October 2023.

Comparison	Groups	Predominant COVID-19 variant period	Number of events per denominator	Unadjusted incidence rate ratio (95% confidence interval)	Adjusted* incidence rate ratio (aIRR) (95% confidence interval)	aIRR ratio *p*-value
Comparison 1	Vaccinated Before v. After Pregnancy	Pre-Delta	32 / 407 ÷ 722 / 13371	1.46 (1.04-2.05)	1.58 (1.13-2.23)	<0.001
	Vaccinated During v. After Pregnancy	Pre-Delta	400 / 9322 ÷ 722 / 13371	0.79 (0.71-0.90)	0.84 (0.75-0.95)	<0.001
	Vaccinated Before v. After Pregnancy	Delta	312 / 6776 ÷ 148 / 1824	0.57 (0.47-0.69)	0.64 (0.53-0.78)	<0.001
	Vaccinated During v. After Pregnancy	Delta	342 / 6909 ÷ 148 / 1824	0.61 (0.51-0.73)	0.64 (0.53-0.77)	<0.001
	Vaccinated Before v. After Pregnancy	Omicron	911 / 19094 ÷ 38 / 344	0.42 (0.31-0.57)	0.48 (0.36-0.66)	<0.001
	Vaccinated During v. After Pregnancy	Omicron	128 / 2559 ÷ 38 / 344	0.44 (0.31-0.62)	0.48 (0.34-0.68)	<0.001
Comparison 2	Vaccinated Before Pregnancy v. Unvaccinated	Pre-Delta	32 / 407 ÷ 6294 / 114334	0.78 (0.71-0.86)	0.85 (0.77-0.94)	<0.001
	Vaccinated During Pregnancy v. Unvaccinated	Pre-Delta	400 / 9322 ÷ 6294 / 114334	1.43 (1.02-1.99)	1.60 (1.15-2.24)	<0.001
	Vaccinated Before Pregnancy v. Unvaccinated	Delta	312 / 6776 ÷ 3282 / 54401	0.82 (0.74-0.91)	0.83 (0.75-0.93)	<0.001
	Vaccinated During Pregnancy v. Unvaccinated	Delta	342 / 6909 ÷ 3282 / 54401	0.76 (0.68-0.85)	0.84 (0.75-0.94)	<0.001
	Vaccinated Before Pregnancy v. Unvaccinated	Omicron	911 / 19094 ÷ 4754 / 79713	0.84 (0.71-1.00)	0.85 (0.71-1.00)	0.057

Vaccinated During Pregnancy v. Unvaccinated	Omicron	128 / 2559 4754 / 79713	0.80 (0.75-0.86)	0.85 (0.79-0.91)	<0.001

*Generated with Poisson regression modeling fitted via generalized estimating equations (GEEs) with robust standard errors, distinguishing vaccination effects across the pre-Delta (prior to June 20, 2021), Delta (on or after June 20, 2021 and before December 26, 2021), and Omicron (on or after December 26, 2021) predominant variant periods, and accounting for data partner site as a clustering variable to account for heterogeneity in EHR-curation and phenotyping processes. Covariates in adjusted models included maternal age at start of pregnancy, race/ethnicity, type of insurance, number of comorbidities (categorical with 7+ as highest level and 0 as referent), record of prior preterm birth (or stillbirth, for respective models in subsequent tables), history of COVID-19 infection prior to current pregnancy, COVID-19 infection during pregnancy (categorical, by trimester), and estimable functions of substance use and/or smoking (vaccination-status/timing and predominant variant period specific predicted values for Gaussian GEE-modeled residuals from a GEE model for the outcome using all other covariates except the near-perfect-predictors[53] of substance use and/or smoking, to adjust for any additional variation in the outcome attributable to substance use and/or smoking as recorded in the data partners' EHR systems).
