# Supplementary material for: Pregnancy Outcomes among Pregnant Persons after COVID-19 Vaccination: Assessing Vaccine Safety in Retrospective Cohort Analysis of U.S. National COVID Cohort Collaborative (N3C)
Source: Vaccines (Basel). 2024 Mar 11;12(3):289. doi: 10.3390/vaccines12030289 (PMC10975285; doi:10.3390/vaccines12030289)
Supplement: Supplementary file 1 [file vaccines-12-00289-s001.zip › Table S4. Unadjusted incidence rate ratios of stillbirth by vaccination status and dominant variant period among pregnant persons.pdf]

Table S4. Unadjusted incidence rate ratios of stillbirth by vaccination status and dominant variant period among pregnant persons in U.S. N3C, December 2020-October 2023.

Comparison	Groups*	Predominant COVID-19 variant period	Number of events** per denominator	Unadjusted incidence rate ratio*** (95% confidence interval)	Unadjusted incidence rate ratio *p*-value
Comparison 1	Vaccinated Before v. After Pregnancy	Pre-Delta	<20 / 410<br>54 / 13245	1.82 (0.36-5.61)	0.477
	Vaccinated During v. After Pregnancy	Pre-Delta	35 / 9357<br>54 / 13245	0.93 (0.59-1.45)	0.825
	Vaccinated Before v. After Pregnancy	Delta	38 / 6814<br><20 / 1836	0.85 (0.44-1.79)	0.736
	Vaccinated During v. After Pregnancy	Delta	27 / 6936<br><20 / 1836	0.60 (0.29-1.29)	0.196
	Vaccinated Before v. After Pregnancy	Omicron	115 / 19209<br><20 / 336	1.01 (0.27-8.4)	1.00
	Vaccinated During v. After Pregnancy	Omicron	<20 / 2570<br><20 / 336	0.72 (0.16-6.68)	0.907
Comparison 2	Vaccinated Before Pregnancy v. Unvaccinated	Pre-Delta	<20 / 410<br>709 / 115043	1.19 (0.24-3.48)	0.927
	Vaccinated During Pregnancy v. Unvaccinated	Pre-Delta	35 / 9357<br>709 / 115043	0.61 (0.42-0.85)	0.003
	Vaccinated Before Pregnancy v. Unvaccinated	Delta	38 / 6814<br>405 / 54806	0.75 (0.54-1.05)	0.105
	Vaccinated During Pregnancy v. Unvaccinated	Delta	27 / 6936<br>405 / 54806	0.53 (0.34-0.78)	0.001
	Vaccinated Before Pregnancy v. Unvaccinated	Omicron	115 / 19209<br>602 / 80315	0.80 (0.65-0.98)	0.027
		Omicron	<20 / 2570	0.57 (0.28-1.03)	0.065

Vaccinated During Pregnancy v. Unvaccinated	602 / 80315

*Nonzero cell counts <20 have been occluded. We use exact inference for unadjusted rates (not adjusting as intended, for covariates and heterogeneity in data partner sites), given smaller sample sizes.

**Nonzero counts <20 are suppressed per N3C Governance guidance to comply with data transfer agreements.

***Generated with unadjusted exact rate estimation,[33] implemented separately for each pair of comparison groups in each of the pre-Delta (prior to June 20, 2021), Delta (on or after June 20, 2021 and before December 26, 2021), and Omicron (on or after December 26, 2021) predominant variant periods. We did not account for data partner sites or covariates in an adjusted model due to current analytic limitations (availability of methods implemented only in commercial software) within the N3C Enclave.
