# Supplementary material for: Pregnancy Outcomes among Pregnant Persons after COVID-19 Vaccination: Assessing Vaccine Safety in Retrospective Cohort Analysis of U.S. National COVID Cohort Collaborative (N3C)
Source: Vaccines (Basel). 2024 Mar 11;12(3):289. doi: 10.3390/vaccines12030289 (PMC10975285; doi:10.3390/vaccines12030289)
Supplement: Supplementary file 1 [file vaccines-12-00289-s001.zip › Table S5. Sensitivity analyses stratifying by COVID-19 infection during pregnancy.pdf]

Table S5: Sensitivity analyses stratifying by COVID-19 infection status during pregnancy in the U.S. N3C, December 2020-October 2023

Table S5a: Unadjusted and adjusted incidence rate ratios (aIRR) of preterm birth during pregnancy by vaccination status and dominant variation period among pregnant persons with documented COVID-19 infection during pregnancy in the U.S. N3C, December 2020-October 2023

Comparison	Groups	Predominant COVID-19 variant period	Number of events per denominator	Unadjusted incidence rate ratio (95% confidence interval)	Adjusted* incidence rate ratio (aIRR) (95% confidence interval)
Comparison 1	Vaccinated Before v. After Pregnancy	Pre-Delta	0 / <20<br>84 / 1521	**0.00 (0.00-4.27)	**_(_-_)
	Vaccinated During v. After Pregnancy	Pre-Delta	30 / 680<br>84 / 1521	0.80 (0.53-1.20)	0.79 (0.53-1.19)
	Vaccinated Before v. After Pregnancy	Delta	56 / 1411<br>27 / 442	0.65 (0.42-1.02)	0.73 (0.47-1.14)
	Vaccinated During v. After Pregnancy	Delta	63 / 1283<br>27 / 442	0.61 (0.51-0.73)	0.84 (0.54-1.31)
	Vaccinated Before v. After Pregnancy	Omicron	202 / 4283<br><20 / 78	0.61 (0.28-1.34)	0.65 (0.31-1.37)
	Vaccinated During v. After Pregnancy	Omicron	<20 / 692<br><20 / 78	0.36 (0.15-0.87)	0.38 (0.16-0.89)
Comparison 2	Vaccinated Before Pregnancy v. Unvaccinated	Pre-Delta	<20 / <20<br>667 / 11991	0.79 (0.55-1.13)	0.81 (0.56-1.16)
	Vaccinated During Pregnancy v. Unvaccinated	Pre-Delta	30 / 680<br>667 / 11991	**0.79 (0.53-1.14)	**_(_-_)
	Vaccinated Before Pregnancy v. Unvaccinated	Delta	56 / 1411<br>699 / 13438	0.94 (0.73-1.21)	0.92 (0.71-1.18)
	Vaccinated During	Delta	63 / 1283	0.76 (0.58-1.00)	0.80 (0.61-1.05)

	Pregnancy v. Unvaccinated		699 / 13438		
	Vaccinated Before Pregnancy v. Unvaccinated	Omicron	202 / 4,283	0.56 (0.36-0.88)	0.55 (0.35-0.86)
			867 / 17816		
	Vaccinated During Pregnancy v. Unvaccinated	Omicron	<20 / 692	0.97 (0.83-1.13)	0.96 (0.83-1.12)
			867 / 17816		

*Generated with Poisson regression modeling fitted via generalized estimating equations (GEEs) with robust standard errors, distinguishing vaccination effects across the pre-Delta (prior to June 20, 2021), Delta (on or after June 20, 2021 and before December 26, 2021), and Omicron (on or after December 26, 2021) predominant variant periods, and accounting for data partner site as a clustering variable to account for heterogeneity in EHR-curation and phenotyping processes. Covariates in adjusted models included maternal age at start of pregnancy, race/ethnicity, type of insurance, number of comorbidities (categorical with 7+ as highest level and 0 as referent), record of prior preterm birth (or stillbirth, for respective models in subsequent tables), history of COVID-19 infection prior to current pregnancy, COVID-19 infection during pregnancy (categorical, by trimester), and estimable functions of substance use and/or smoking (vaccination-status/timing and predominant variant period specific predicted values for Gaussian GEE-modeled residuals from a GEE model for the outcome using all other covariates except the near-perfect-predictors[53] of substance use and/or smoking, to adjust for any additional variation in the outcome attributable to substance use and/or smoking as recorded in the data partners' EHR systems).
**Nonzero cell counts <20, along with corresponding adjusted-using-large-sample-reliant regression methods, have been occluded. We use exact inference for unadjusted rates (not adjusting as intended, for covariates and heterogeneity in data partner sites), given smaller sample sizes in these instances.

Table S5b: Unadjusted and adjusted incidence rate ratios (aIRR) of preterm birth during pregnancy by vaccination status and dominant variation period among pregnant persons with no documented COVID-19 infection during pregnancy in the U.S. N3C, December 2020-October 2023

Comparison	Groups	Predominant COVID-19 variant period	Number of events per denominator	Unadjusted incidence rate ratio (95% confidence interval)	Adjusted* incidence rate ratio (aIRR) (95% confidence interval)
Comparison 1	Vaccinated Before v. After Pregnancy	Pre-Delta	32 / 391	1.52 (1.08-2.14)	1.63 (1.16-2.29)
			638 / 11850		
	Vaccinated During v. After Pregnancy	Pre-Delta	370 / 8642	0.80 (0.70-0.90)	0.84 (0.74-0.95)
			638 / 11850		
	Vaccinated Before v. After Pregnancy	Delta	256 / 5365	0.54 (0.43-0.67)	0.61 (0.50-0.75)
			121 / 1382		
	Vaccinated During v. After Pregnancy	Delta	279 / 5626	0.57 (0.46-0.70)	0.59 (0.48-0.73)
			121 / 1382		
	Vaccinated Before v. After Pregnancy	Omicron	709 / 14811	0.38 (0.27-0.53)	0.44 (0.31-0.62)
			32 / 256		
	Vaccinated During v. After Pregnancy	Omicron	109 / 1867	0.47 (0.32-0.68)	0.50 (0.34-0.74)
			32 / 256		
Comparison 2	Vaccinated Before Pregnancy v. Unvaccinated	Pre-Delta	32 / 391	0.78 (0.70-0.86)	0.85 (0.77-0.95)
			5627 / 102343		
	Vaccinated During Pregnancy v. Unvaccinated	Pre-Delta	370 / 8642	1.49 (1.07-2.08)	1.66 (1.19-2.32)
			6294 / 114334		
	Vaccinated Before Pregnancy v. Unvaccinated	Delta	256 / 5365	0.79 (0.70-0.89)	0.81 (0.72-0.92)
			2583 / 40963		
	Vaccinated During	Delta	279 / 5626	0.76 (0.67-0.86)	0.84 (0.74-0.95)

	Pregnancy v. Unvaccinated		2583 / 40963		
	Vaccinated Before Pregnancy v. Unvaccinated	Omicron	709 / 14811	0.93 (0.77-1.12)	0.94 (0.78-1.13)
			3887 / 61897		
	Vaccinated During Pregnancy v. Unvaccinated	Omicron	109 / 1867	0.76 (0.71-0.82)	0.82 (0.76-0.89)
			3887 / 61897		

*Generated with Poisson regression modeling fitted via generalized estimating equations (GEEs) with robust standard errors, distinguishing vaccination effects across the pre-Delta (prior to June 20, 2021), Delta (on or after June 20, 2021 and before December 26, 2021), and Omicron (on or after December 26, 2021) predominant variant periods, and accounting for data partner site as a clustering variable to account for heterogeneity in EHR-curation and phenotyping processes. Covariates in adjusted models included maternal age at start of pregnancy, race/ethnicity, type of insurance, number of comorbidities (categorical with 7+ as highest level and 0 as referent), record of prior preterm birth (or stillbirth, for respective models in subsequent tables), history of COVID-19 infection prior to current pregnancy, COVID-19 infection during pregnancy (categorical, by trimester), and estimable functions of substance use and/or smoking (vaccination-status/timing and predominant variant period specific predicted values for Gaussian GEE-modeled residuals from a GEE model for the outcome using all other covariates except the near-perfect-predictors[53] of substance use and/or smoking, to adjust for any additional variation in the outcome attributable to substance use and/or smoking as recorded in the data partners' EHR systems).

Table S5c: Unadjusted incidence rate ratios (IRR) of stillbirth during pregnancy by vaccination status and dominant variation period among pregnant persons with documented COVID-19 infection during pregnancy in the U.S. N3C, December 2020-October 2023

Comparison	Groups*	Predominant COVID-19 variant period	Number of events** per denominator	Unadjusted incidence rate ratio*** (95% confidence interval)
Comparison 1	Vaccinated Before v. After Pregnancy	Pre-Delta	0 / <20	0.00 (0.00-48.45)
			<20 / 1530	
	Vaccinated During v. After Pregnancy	Pre-Delta	<20 / 681	0.25 (0.01-1.80)
			<20 / 1530	
	Vaccinated Before v. After Pregnancy	Delta	<20 / 1418	1.10 (0.21-10.81)
			<20 / 444	
	Vaccinated During v. After Pregnancy	Delta	<20 / 1286	0.52 (0.06-6.20)
			<20 / 444	
	Vaccinated Before v. After Pregnancy	Omicron	20 / 4303	∞ (0.089 - ∞)
			0 / 78	
	Vaccinated During v. After Pregnancy	Omicron	<20 / 693	∞ (0.003 - ∞)
			0 / 78	
Comparison 2	Vaccinated Before Pregnancy v. Unvaccinated	Pre-Delta	0 / <20	0 (0.00 - 29.28)
			97/ 12088	
	Vaccinated During Pregnancy v. Unvaccinated	Pre-Delta	<20 / 681	0.18 (0.005-1.04)
			97/ 12088	
	Vaccinated Before Pregnancy v. Unvaccinated	Delta	<20 / 1418	0.81 (0.32-1.75)
			82 / 13520	
	Vaccinated During Pregnancy v.	Delta	<20 / 1286	0.38 (0.08-1.16)

	Unvaccinated		82 / 13520	
	Vaccinated Before Pregnancy v. Unvaccinated	Omicron	20 / 4303	1.00 (0.58-1.65)
			83/ 17899	
	Vaccinated During Pregnancy v. Unvaccinated	Omicron	<20 / 693	0.31 (0.008-1.78)
			83/ 17899	

*Nonzero cell counts <20 have been occluded. We use exact inference for unadjusted rates (not adjusting as intended, for covariates and heterogeneity in data partner sites), given smaller sample sizes.
**Nonzero counts <20 are suppressed per N3C Governance guidance to comply with data transfer agreements.
***Generated with unadjusted exact rate estimation,[33] implemented separately for each pair of comparison groups in each of the pre-Delta (prior to June 20, 2021), Delta (on or after June 20, 2021 and before December 26, 2021), and Omicron (on or after December 26, 2021) predominant variant periods. We did not account for data partner sites or covariates in an adjusted model due to current analytic limitations (availability of methods implemented only in commercial software) within the N3C Enclave.

Table S5d: Unadjusted incidence rate ratios (aIRR) of stillbirth during pregnancy by vaccination status and dominant variation period among pregnant persons with no documented COVID-19 infection during pregnancy in the U.S. N3C, December 2020-October 2023

Comparison	Groups*	Predominant COVID-19 variant period	Number of events** per denominator	Unadjusted incidence rate ratio*** (95% confidence interval)
Comparison 1	Vaccinated Before v. After Pregnancy	Pre-Delta	<20 / 394	2.01 (0.63-6.45)
			45 / 11895	
	Vaccinated During v. After Pregnancy	Pre-Delta	34 / 8676	1.04 (0.66-1.62)
			45 / 11895	
	Vaccinated Before v. After Pregnancy	Delta	31 / 5396	0.80 (0.39-1.63)
			<20 / 1392	
	Vaccinated During v. After Pregnancy	Delta	24 / 5650	0.59 (0.28-1.23)
			<20 / 1392	
	Vaccinated Before v. After Pregnancy	Omicron	95 / 14906	0.82 (0.20-3.32)
			<20 / 258	
	Vaccinated During v. After Pregnancy	Omicron	<20 / 1877	0.69 (0.15-3.12)
			<20 / 258	
Comparison 2	Vaccinated Before Pregnancy v. Unvaccinated	Pre-Delta	<20 / 394	2.01 (0.40-6.27)
			612 / 102955	
	Vaccinated During Pregnancy v. Unvaccinated	Pre-Delta	34 / 8676	1.04 (0.64-1.65)
			612 / 102955	
	Vaccinated Before Pregnancy v. Unvaccinated	Delta	31 / 5396	0.8 (0.38-1.83)
			323 / 41286	
	Vaccinated During Pregnancy v.	Delta	24 / 5650	0.59 (0.27-1.39)

	Unvaccinated		323 / 41286	
	Vaccinated Before Pregnancy v. Unvaccinated	Omicron	95 / 14906	0.82 (0.22-6.89)
			519 / 62416	
	Vaccinated During Pregnancy v. Unvaccinated	Omicron	<20 / 1877	0.69 (0.15-6.45)
			519 / 62416	

*Nonzero cell counts <20 have been occluded. We use exact inference for unadjusted rates (not adjusting as intended, for covariates and heterogeneity in data partner sites), given smaller sample sizes.
**Nonzero counts <20 are suppressed per N3C Governance guidance to comply with data transfer agreements.
***Generated with unadjusted exact rate estimation,[33] implemented separately for each pair of comparison groups in each of the pre-Delta (prior to June 20, 2021), Delta (on or after June 20, 2021 and before December 26, 2021), and Omicron (on or after December 26, 2021) predominant variant periods. We did not account for data partner sites or covariates in an adjusted model due to current analytic limitations (availability of methods implemented only in commercial software) within the N3C Enclave.
